# Identification and Distribution of Begomoviruses Infecting Cassava Fields in Sierra Leone

**DOI:** 10.3390/plants14142142

**Published:** 2025-07-11

**Authors:** Musa Decius Saffa, Alusaine Edward Samura, Mohamed Alieu Bah, Angela Obiageli Eni, Ezechiel B. Tibiri, Saïdou Zongo, William J.-L. Amoakon, Fidèle Tiendrébéogo, Justin Simon Pita, Prince Emmanuel Norman

**Affiliations:** 1 Department of Crop Protection, School of Agriculture and Food Sciences, Njala University, Njala Campus, Private Mail Bag, Njala, Sierra Leone; alusaine.samura@wave-center.org; 2 Department of Crop Science, School of Agriculture and Food Sciences, Njala University, Njala Campus, Private Mail Bag, Njala, Sierra Leone; abah@njala.edu.sl; 3 Regional Center of Excellence for Transboundary Plant Pathogens, Central and West African Virus Epidemiology (WAVE), Pôle Scientifique et d’Innovation, Université Félix Houphouët-Boigny, Abidjan BPV 34, Côte d’Ivoire; angela.eni@wave-center.org (A.O.E.); willamoakon@gmail.com (W.J.-L.A.); justin.pita@wave-center.org (J.S.P.); 4 Laboratoire de Virologie et de Biotechnologie Végétale (LVBV), Institut de l’Environnement et de Recherches Agricoles (INERA), Ouagadougou 04 BP 8645, Burkina Faso; ezechiel.tibiri@ujkz.bf (E.B.T.); zongosaidou642@gmail.com (S.Z.); 5 Germplasm Enhancement and Seeds System, Sierra Leone Agricultural Research Institute (SLARI), Tower Hill, PMB 1313, Freetown, Sierra Leone; norman.prince64@gmail.com

**Keywords:** epidemiology, viruses, East African cassava mosaic (EACMV), African cassava mosaic virus (ACMV), *Manihot esculenta*

## Abstract

A dearth of knowledge exists on identifying the begomoviruses and distributing cassava mosaic viruses across key cassava-growing regions of Sierra Leone. The study aimed to identify and map the distribution of cassava mosaic disease (CMD)-associated viruses in farmers’ fields in Sierra Leone. Cassava (*Manihot esculenta* Crantz) leaf samples were collected in 109 smallholder farms during a geo-referenced survey conducted from 10th May to 5th June 2024. Molecular diagnostics were carried out to identify the viral strains associated with CMD. Findings revealed that infection by stem cutting was more predominant in the south, east, north, and northwest regions than in the west region. In contrast, infection by whitefly was predominant in the west, north, and northwest regions. PCR screening of 426 samples coupled with sequence analysis revealed the presence of African cassava mosaic-like (ACMV-like) viruses, and East African cassava mosaic-like (EACMV-like) viruses as single infections at 78.1% and 1.3%, respectively. Co-infections of ACMV-like and EACMV-like viruses were detected in 20.6% of the tested samples. In addition, 70.6% of the samples positive for EACMV-like virus (single and mixed infections) were found to be positive for East African cassava mosaic Cameroon virus (EACMCMV). The ACMV and co-infection of ACMV and EACMV viruses were present in all regions, while EACMCV was detected in all regions except the western area. The results indicate more prevalence of the EACMCMV variant in Sierra Leone. This study suggests utilization of participatory surveillance and good agronomic practices to manage CMD in Sierra Leone.

## 1. Introduction

Cassava (*Manihot esculenta* Crantz) is grown in many countries in Sub-Saharan Africa where it supports the livelihoods of over 500 million people as a source of food, feed, and income and in various industrial applications [1]. Africa accounts for >80% of the world’s cassava-cultivated land [2]. However, farmers do not fully benefit from the crop’s yield potential due to the many pests and diseases, and poor agricultural management practices resulting from a lack of the resources and knowledge to practice profitable and sustainable farming [3]. The most devastating diseases detected are cassava mosaic disease (CMD) and cassava brown streak disease (CBSD), which are caused by viruses [4]. These diseases are considered to be among key biotic constraints of cassava production in the 21st century [5]. CMD is present in all cassava-growing regions in Africa and can account for >80% of cassava fresh storage root yield losses [6]. According to [7], CMD accounts for more than 30 million tons of fresh storage root yield losses of cassava annually. African cassava mosaic virus and East African cassava mosaic virus (ACMV-EACMV) co-infection can cause up to 82% crop loss in Africa [5]. In Sub-Saharan Africa, the East African cassava mosaic Cameroon virus, East African cassava mosaic Malawi virus, East African cassava mosaic Zanzibar virus, Southern Africa cassava mosaic virus, EACMV, ACMV, and Indian cassava mosaic virus (ICMV) have been noted to cause devastating damage in cassava fields [8]. Crop losses by EACMV are estimated at 68%, and ACMV can reach up to 30–40%. In West Africa, high CMD infection can reduce cassava storage root yield by up to 90% if control intervention is not implemented [9]. CMD is spread by whitefly vectors (*Bemisia tabaci*) and infected stem cuttings [10,11,12]. The disease is widespread in several east, central, west, and southern African countries [13,14,15].

Cassava mosaic disease is caused by a cassava mosaic begomoviruses (CMBs) complex, which constitute 11 species of bipartite begomoviruses, of which 9 have been detected in Africa [16,17]. The distribution and spread of these begomoviruses often differ from one country to another and/or from one region to another within the same country [18,19,20,21,22]. Cassava begomovirus, known as alternate hosts, can infect the primary and secondary hosts, cassava, and other weed species that grow around cassava fields. This makes managing the virus and eradicating it highly challenging.

The disease situation in the cassava fields is exacerbated by the fact that the crop is traditionally cultivated using the stem cutting technique. Cassava propagation through stem cuttings is among the constraints of producing disease-free planting materials due to high accumulation of diseases during successive production cycles [23]. Cassava botanical seed materials generally exhibit slow germination due to dormancy. The accumulation of viral, fungal, and bacterial diseases in the stem cuttings contributes to decreased cassava production and productivity, leading to the loss of superior genotypes [24]. Moreover, diseases and pests affect the growth and development of cassava, consequently contributing to reduced economic yields in several production zones in Africa [25,26].

In Sierra Leone, several epidemiological studies have detected CMD in most cassava-growing areas [9,27,28,29]. These studies have focused on screening for resistance in different agroecosystems, incidence, and severity of CMD, and evaluating of impact of the disease on cassava genotypes in the country. Despite these various studies in Sierra Leone, the identification and/or characterization of the begomoviruses and distribution of cassava mosaic viruses remains less documented in several provinces. The present study aimed to identify and map the distribution of CMD-associated viruses in farmers’ fields in Sierra Leone.

## 2. Results

### 2.1. Phenotypic Assessment of Cassava Mosaic Disease

**Figure 1:**
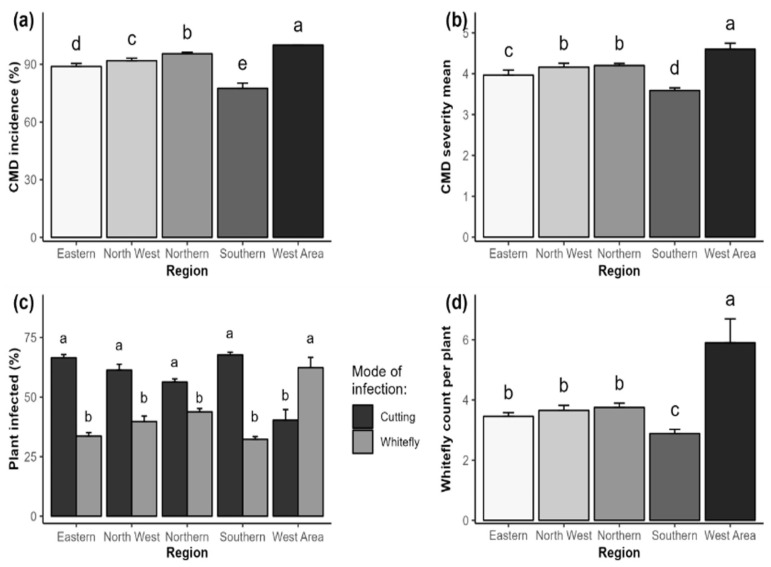
Epidemiological assessment of cassava mosaic disease (CMD) across regions of Sierra Leone. (**a**) Mean incidence of CMD, (**b**) mean severity of CMD, (**c**) percent of cuttings or whitefly-infected plants, (**d**) mean whiteflies per plant. Error bars represent standard error (SE). abcde in the figure represent significant variation among treatments for the parameter or trait measured.

**Figure 2:**
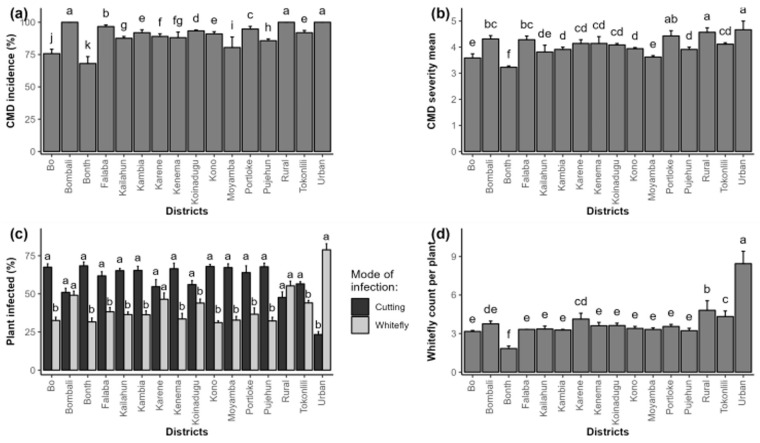
Epidemiological assessment of cassava mosaic disease (CMD) per district in Sierra Leone. (**a**) Mean incidence of CMD, (**b**) mean severity of CMD, (**c**) percent cuttings or whitefly-infected plants, (**d**) mean whiteflies per plant. Error bars represent standard error (SE). abcdeghijk in the figure represent significant variation among treatments for the parameter or trait measured.

Generally, the mean incidence and severity of CMD, percent of plants infected by cuttings or whiteflies, and number of whiteflies per plant significantly (*p* < 0.05) varied among the regions and districts of Sierra Leone (Figure 1 and Figure 2). CMD symptoms were found in all 109 cassava fields surveyed, with the highest incidence and severity recorded in the west region. In contrast, the south region recorded the lowest values (Figure 1a,b). The percent of infected plants through stem cutting was more predominant in the south, east, north, and northwest regions than in the west region, where the whitefly mode of infection was higher than the stem cutting (Figure 1c). The highest whitefly abundance was recorded in the western region, with the lowest proportion recorded in the southern region (Figure 1d).

At the district level, the highest CMD incidence scores of almost 100% were recorded in Bombali, western rural and western urban districts, with the lowest of 70% CMD incidence found in the Bonthe district. The western rural and western urban districts exhibited the highest severity values of 4.8 (high infection), with the lowest of 3.2 (low infection of the disease) recorded in the Bonthe district (Figure 2a,b). The percentage of infected plants through stem cutting was more predominant in all the districts except western rural and western urban districts, where the whitefly mode of infection was higher than the stem cutting (Figure 2c). The highest whitefly abundance was captured in the western urban district, followed by the western rural district, with the lowest of two whiteflies per plant recorded in the Bonthe district (Figure 2d).

### 2.2. Phenotypic and Molecular Detection and Distribution of Cassava Begomoviruses in Sierra Leone

**Figure 3:**
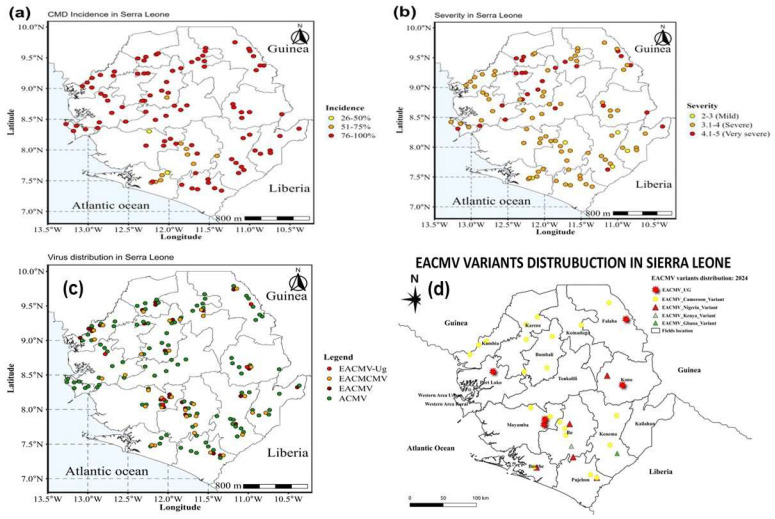
Maps of showing (**a**) distribution of cassava mosaic disease (CMD) incidence; (**b**) distribution of severity of CMD; (**c**) distribution of type of cassava mosaic disease infection; and (**d**) distribution of east African cassava mosaic virus variants in Sierra Leone.

The distribution of CMD infection in cassava fields of Sierra Leone is also shown in distribution maps (Figure 3). The results reveal that the incidence of CMD ranged from 25 to 50% in the south region (Moyamba, Bonthe, and Bo districts) and some parts of the Bombali district, followed by some farms with 51–75% incidence of CMD found in the north and northwest regions. The east, north, northwest, and west areas recorded the highest incidence of CMD, ranging from 76 to 100%. The south region recorded the lowest severity scores of 2.0–3.0 (mild) and 3.1–4.0 (severe) infection of the disease. The Kailahun and Kono districts in the east region had severity scores of 3.1–4.0 and 4.1–5.0, respectively. In the north region, cassava farms in the Falaba and Bombali districts recorded severity scores of 3.1–4.0 and 4.1–5.0, respectively. Karene in the northwest region and the western rural and western urban districts in the west region had the highest severity damage, ranging from 4.1 to 5.0.

The distribution map of the CMBs (ACMV, EACMV, EACMCMV, and EACMV-Ug) detected in this study is shown in Figure 3c. The results revealed that 5 out of the 426 samples screened were infected by EACMV-Ug. These EACMV-Ug-infected samples were detected in cassava farms in the Moyamba, Port Loke, Falaba, and Kono districts. These districts share a border with Guinea, except the Moyamba district.

A search for related sequences in the GenBank database (NCBI, BLASTN) revealed the existence and spread of EACMV_Ug in four districts with red-like star geo-reference points (Figure 3d). Sequences of 40 cassava samples detected as positive for the ACMV were most closely related to ACMV, EACMV_Ug, EACMVCM, EACMV_Nigeria, and EACMV_Ghana virus. The EACMVCM virus is widely spread across the country, with geo-reference points in yellow, and is distributed mainly in the south and north regions of Sierra Leone. The blast results showed few EACMV Nigeria, Ghana, and Kenyan variants.

For PCR analysis, 426 cassava leaf samples were collected from 320 plants with and 106 without symptoms in 2024. Among the samples with observable symptoms, 5.6% (28/320) tested negative for ACMV and EACMV. On the other hand, 12.3% (13/106) and 5.7% (6/106) of symptomless samples tested positive for ACMV and mixed infection of ACMV and EACMV, respectively. Of the 426 samples, 311 (73.0%) were found positive, with 115 (27.0%) recorded as unfavorable (Table 1 and Table 2). Among the positive samples studied, the single ACMV infection was the most frequent, accounting for 78.1% (243/311) of all CMD begomovirus infections, followed by mixed infections of ACMV and EACMV with 20.6% (64/311), and single infection of EACMV with 1.3% (4/311).

**Figure 4:**
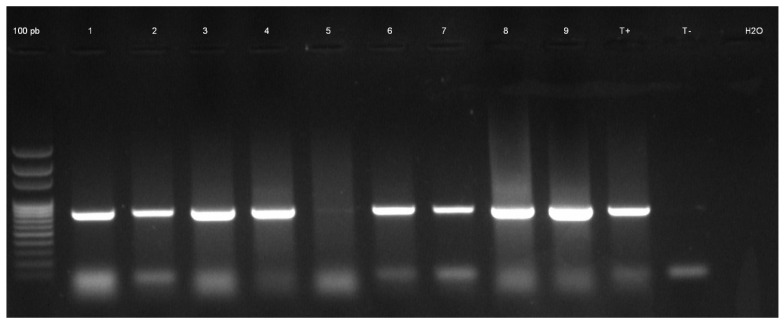
PCR amplification of cassava DNA using JSP001/JSP002. ACMV M = DNA ladder, ACMV detection at 783 bp = lanes 1–4, and 6–9; positive control = lane T^+^; negative control = lane T^−^; and water = lane H_2_O.

The typical gel electrophoresis photo of ACMV begomoviruses detected in the study is presented in Figure 4.

The single infection of ACMV predominated in all surveyed regions, with the highest proportion (93.1%, 27/29) in the western area, followed by the north region (87.7%, 71/81), whereas the south had the lowest of 64.4% (47/73). Mixed infection occurred in all the regions, with the highest proportions in south (35.6%, 26/73), and the lowest of 6.9% (2/29) was detected in the western area. Single infection of EACMV was found in the northwest (5.0%, 3/60) and east (13.2%, 1/68) regions, whereas single infection of EACMV was not detected in the regions of the south, north, and western areas (Table 2). Of the 68 EACMV positive samples (single and mixed infections), 70.6% (48/68) tested positive for EACMVCM using the primer pair VNF031/VNF032.

**Figure 5:**
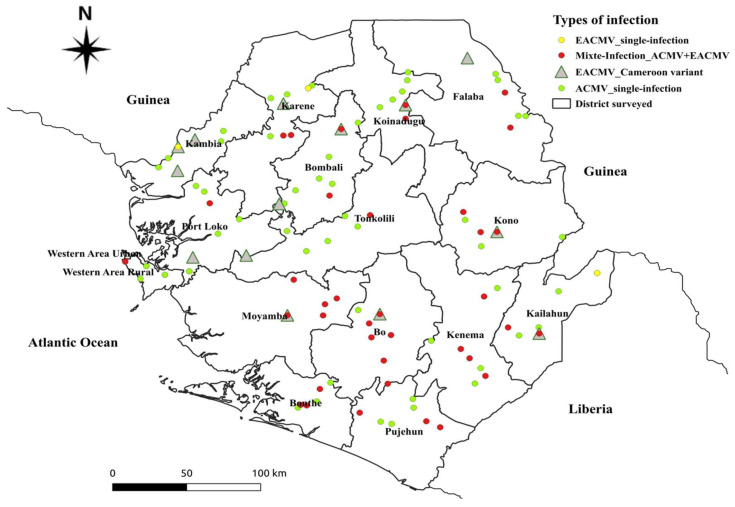
Distribution of cassava mosaic begomoviruses isolates in study areas of Sierra Leone.

Figure 5 show the distribution of cassava mosaic begomoviruses detected in smallholder cassava farmers’ fields of Sierra Leone. The ACMV single infection is widely spread across the districts of Sierra Leone represented by green geo-reference points, followed by ACMV/EACMV mix infection (with red geo-reference points), which is widely spread particularly in the south and north regions. The EACMVCM infection, illustrated by a gray color triangular shape, is widely spread in the northwest region and some parts in the south and east regions, while the EACMV single infection is only found in the east and the northwest regions with geo-reference points in yellow.

## 3. Discussion

The highest incidence rates of CMD in the east, north, northwest, and west areas recorded during the 2024 survey suggest that use of infected planting material was probably the cause of the spread of CMD in cassava fields. Farmers have traditionally reused cuttings from their own fields as planting material and these cuttings are often infected with viruses. This leads to the perpetuation of viruses via infected cuttings [30,31]. Similar trends were detected for CMD in several countries in Sub-Saharan Africa, indicating that CMD symptoms observed depend on virus species, strains, and mixed infections [32,33].

The mode of disease infection in cassava fields reveals higher whitefly-transmitted CMD prevalence relative to the cutting-borne CMD in western urban areas partly due to genetic, environmental, cultivation, management, and utilization factors. *Bemisia tabaci* (whitefly) is a polyphagous, highly destructive pest that is capable of vectoring viruses in most agricultural crops including cassava. In the western area, most of the farmers predominantly grow local cassava varieties mainly for foliage utilized as vegetable in cassava sauce. As such, a higher prevalence of whiteflies exists in farmers’ fields with young cassava plantations relative to other regions where cassava plants can stay up to 12 months after planting. The number of whiteflies (*B. tabaci*) on cassava plants may not always match field disease incidence, indicating the existing probable causal agents more than the resultant CMD incidence on cassava plants. These findings corroborate the view that field disease incidence is not always linked with the number of whiteflies on cassava plants [34]. In the present study, the western area is situated around the coastal area, which may also have played a role in the pest and disease dynamics, as is similar to the findings of Njoroge et al. [35]. Thus, higher whitefly density on cassava does not always result in higher cassava virus disease incidence.

The detection and confirmation of EACMV-Ug and its spread in four different districts in Sierra Leone seriously threaten cassava production in the country. This virus was first detected in Forécariah, Guinea, located near the border of Sierra Leone and in the Kambia district, north region of Sierra Leone [36]. Kambia is about 11 km from the Guinean border, whereas Forécariah is 34.8 km from the Sierra Leone border. Considering trade and high traffic activities between this border town of Forécariah, Guinea and Sierra Leone, it is probable that EACMV-Ug was introduced into the country from Forécariah through the exchange of cassava planting materials. The EACMV-Ug virus was found in single and mixed infections with ACMV. Several authors opined that co-infection of ACMV and EACMV-Ug could result in a synergistic interaction that cause epidemics and has a severe impact on cassava, as typified by the incidence in East Africa during the 1990s [37,38,39]. Accordingly, the blast analysis of the full-length nucleotide sequences of EACMV-Ug revealed trans-replication between the DNA molecules circulating in Guinea and Sierra Leone, which are very similar to EACMV-Ug2 DNA-A and EACMV-Ug3 DNA-B described in Uganda [37]. The reassortant virus resulting from this association is a severe EACMV-Ug variant favored over the existing less severe EACMV-Ug1 strain. This reassortant virus causes very severe CMD symptoms. The analytical observations depicted close relatedness of the EAMCV-Ug strain from Guinea and Sierra Leone to the one discovered during the Ugandan epidemic. This emerging biotic constraint is a threat to the economy and resource-poor cassava growers of Sierra Leone, considering the devastation caused by EACMV-Ug in cassava fields in Uganda during the 1990s epidemic.

The present epidemiological study revealed that CMD single incidence varied greatly amongst regions. The highest CMD single infections were observed in four districts in the north region, which indicate the spreading risk potentialities of CMD to new areas. This assertion is in conformity with [40], who opined that disease spread is largely attributable to the planting material exchange by cassava producers without following due procedures of ascertaining their phytosanitary status. The lowest CMD detection found in four districts of the south region is possibly attributable to the high adoption and utilization of improved cassava varieties resistant to CMD [41]. Findings of the molecular detection analytical technique based on the samples collected during the 2024 survey in Sierra Leone showed that the country is increasing its hotspot potentiality of cassava begomoviruses diversity in the West African region. Indeed, four begomoviruses, including ACMV, EACMV, EACMCMV, and EACMV-Ug, were previously found in single, double, and triple infections in Guinea and Sierra Leone [35]. This is the second report on such multiple associations of cassava mosaic begomoviruses in cassava plants in Sierra Leone because a recent publication has shown the Uganda virus incident in Sierra Leone [36]. It reflects the importance of CMD pressure on cassava production in Sierra Leone and raises serious concerns about the origin of the diverse CMBs detected in this country. The ACMV was the most detected disease in all the five regions studied in Sierra Leone, as reported for almost all the Sub-Saharan African countries where CMD occurs [42].

Although triple infections were found in all the regions in Sierra Leone, the southeast and northwest regions registered the highest number of plants infected by ACMV + EACMCMV + EACMV-Ug associated with very severe CMD. The synergistic action among the three viruses involved in this triple co-infection probably contributed to the increased symptom severity, as mentioned by Harimalala et al. [41]. In Burundi, triple infections, ACMV + EACMV + EACMV-Ug, were also detected [43]. In Guinea, EACMV-Ug was detected in four regions, including the one sharing a border with Sierra Leone [36]. An alarming southeast and northwest spread of EACMV-Ug in four different districts in Sierra Leone is similar to the southward spread in Uganda during the CMD epidemic mentioned in several studies [37,43,44]. The blast analysis of EACMV-Ug DNA-A sequences from Sierra Leone showed close relatedness to those from East and Central Africa. Moreover, the EACMV-Ug sequences from Guinea and Sierra Leone contain the same ACMV recombinant fragment in their coat protein (CP) indicating that it is the same reassortant virus EACMV-Ug2 DNA-A + EACMV-Ug3 DNA-B that is circulating in these countries. Findings suggest that this virus was probably introduced into Guinea from east or central African countries via infected cassava planting materials.

Most of the cassava varieties cultivated in Sierra Leone are local varieties such as cocoa, cooksoon, pink lady, blue boat, etc. These varieties are susceptible to CMD with varying degrees. These results advocate for the region’s urgent deployment of CMD management strategies and CMD-resistant varieties. Phytosanitation is a critical component of mitigating the distribution of CMD in Sierra Leone. The main features of this technique include the following: the use of resistant CMD genotypes; removal of diseased cassava from within and immediately around areas to be used for new plantings; the removal (rogueing) of diseased plants from within crop stands; and the use of CMD-free stem cuttings as planting material. Resistant genotypes exhibit lower concentrations of CMD than susceptible ones, as demonstrated serologically by Fargene et al. [45]. This suggests that resistant genotypes are poor sources of inoculum from which vectors can acquire and transmit viruses to uninfected plants. Thus, the adoption of such genotypes contributes to a decrease in the amount of inoculum present in the cassava farming communities or regions and restricts the spread of CMD to any susceptible genotypes being cultivated. The probable consequence is that strands of virus-resistant genotypes sustain little damage or yield loss due to CMD, even under conditions of high inoculum pressure since only a proportion of the plants are infected and those infected sustain little damage and spread. Moreover, CMD occurs in only a proportion of the cuttings collected from infected plants of resistant genotypes so that the incidence of CMD in the cuttings is usually less than in the stand from which they were collected. It is probable that this ‘cleansing’ effect is further enhanced if infection decreases vegetative vigor, or farmers discriminate in favor of vigorous and/or symptomless plants when selecting cuttings for cultivation. For these reasons the incidence of infection is unlikely to increase progressively in successive cycles of propagation, as might otherwise be expected with a vector-borne virus of a vegetatively propagated crop. Rogueing is one of the official schemes for the selection and maintenance of CMD-free stocks for release to farmers. However, this technique may lead to a progressive and unacceptable decrease in stand in areas of high inoculum pressure where much spread occurs, suggesting that rogueing has little effect on reducing the spread within treated fields. Rogueing is probably most effective when practiced by groups of farmers or throughout whole cassava farming communities. The use of CMD-free stem cuttings as planting material could be imperative since cassava plants, including those affected by CMD, regenerate readily from stems left in or on the ground at harvest. Moreover, farmers often harvest piecemeal and from the most vigorous unaffected plants within a stand and then establish new cuttings in the gaps created. This means that young plants often develop beneath or immediately alongside older infected ones. These are potential sources of virus inoculum and also of other pathogens and pests including the cassava green mite (*Mononychehs tanajoa* Bondar) and the cassava mealybug (*Phenacoccus manihoti* Matile-Ferrero). One of the novel techniques utilized for the rapid generation of CMD-free stem cuttings is semi-autotrophic hydroponics (SAHs). The technique utilizes virus-indexed plantlets from tissue culture as starter materials.

## 4. Materials and Methods

### 4.1. Surveyed Experimental Sites

**Figure 6:**
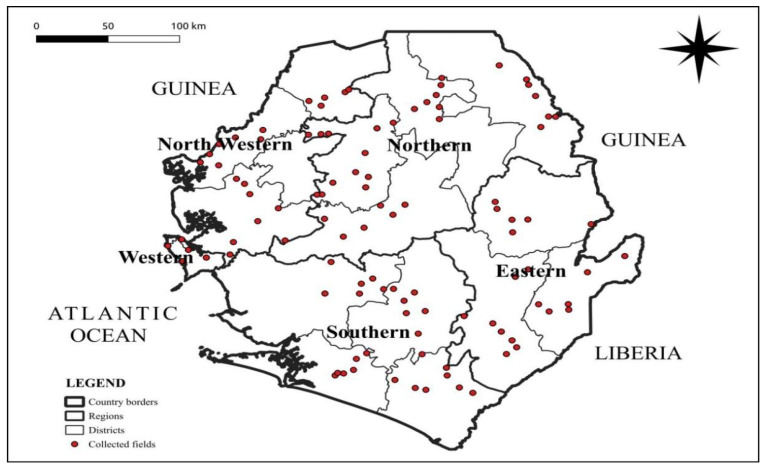
Map of study area showing country borders, regions, districts, and fields.

Sierra Leone, a country located on the west coast of Africa between latitudes 7° and 10° N and longitudes 10° and 13° W, offers a rich and complex field of study encompassing disciplines such as history, political science, anthropology, economics, environmental studies, and public health. Covering a total land area of approximately 71,740 km^2^, Sierra Leone boasts a diverse landscape with a wide array of geographical features important for survey studies. The western region features flat coastal plains extending inland for about 100 km, characterized by mangrove swamps and river deltas. As one moves inland, the terrain becomes more rugged, with the central and eastern parts of the country dominated by hills and mountains. The Loma Mountains, including the highest peak, Mount Bintumani, at 1945 m, provide significant elevation. Dense tropical rainforests cover much of the eastern region, contributing to biodiversity and ecological studies. Sierra Leone borders Guinea to the north and northeast, with a border length of approximately 652 km, and Liberia to the southeast, with a border length of about 306 km. The Atlantic Ocean forms the western boundary, providing a coastline of about 402 km, critical for marine and coastal surveys. The country experiences a tropical climate with a wet season from May to November characterized by heavy rains, receiving annual precipitation ranging between 2000 and 3000 mm, with the coastal areas receiving the highest amounts. From December to April, the dry season is marked by lower precipitation and harmattan winds, affecting visibility and survey conditions. The map of the study area showing the regions, districts, and cassava farms is presented in Figure 6.

### 4.2. Survey Design

A cassava mosaic disease assessment survey was conducted in 2024 using the Central and West African Virus Epidemiology (WAVE) harmonized sampling and diagnostic protocols [42]. Assessments were carried out on 109 farms covering five regions, sixteen districts, and agro-ecologies (rain forest, coastal plains, savannah lowlands, and savannah highlands) of Sierra Leone. The Cassava fields surveyed were 10 km apart. At each farm, 30 cassava plants were randomly selected and evaluated along two diagonals. The geolocation coordinates of each field were recorded using a global positioning device (Garmin Dakota TM 10 Garmin Limited, St. Olathe, KS, USA).

### 4.3. Field Data Collection and Storage

A tablet with iForm Zerion (version 9.1.6) software developed by Cambridge, UK’s Epidemiology modeling group for surveying, was used for data collection in all West African virus epidemiology participating countries. The data collected included the name of the village or town, the district, region, whitefly counts, cassava mosaic disease symptom observed, geographical coordinates (latitude and longitude), mode of infection, and altitude. Data were collected on variety, date and time, field size, planting types, and distance between survey sites. The recorded data were uploaded to iForm’s cloud-based database and integrated into the WAVE Cube. The data collected were CMD severity, whitefly abundance, and mode of infection (i.e., either cuttings or vector). According to Sseruwagi et al. [15], a distinction between cutting-borne and whitefly-borne infections is possible from three to six MAPs. Symptoms found only on the upper leaves of plants were considered to have resulted from whitefly-transmitted infection, whereas symptoms on the lower leaves or on all leaves were taken as having been infected through cassava cuttings.

**Figure 7:**
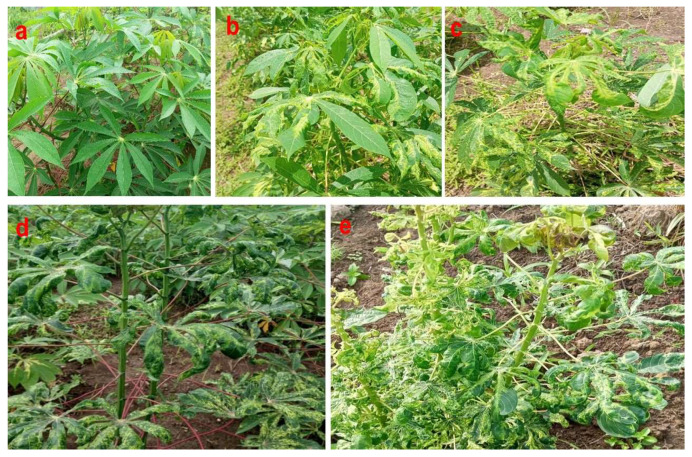
Cassava showing (**a**) healthy-looking plant; (**b**–**e**) cassava mosaic disease (CMD)-infected plants.

The cassava disease severity evaluation was calculated using the 1–5 disease rating scale (Figure 7), where 1 = symptomless plants; 2 = mild chlorotic patterns affecting most leaves, mild distortions at the bases of most leaves and remaining part of the leaves are typical; 3 = pronounced chlorosis on most leaves, narrowing and distortion of the lower one-third of the leaflets; 4 = severe chlorosis and distortion of two-thirds of most leaves and general reduction in leaf size and some stunting; and 5 = most severe symptoms (severe chlorosis, leaf distortion, twisting, misshapen leaves, severe reduction in most leaves, and severe plant stunting) [46]. CMD incidence was calculated as the quotient of CMD symptomatic plants over the total number of plants expressed as a percentage following the formula by Sseruwagi et al. [15].

Mean incidence (%) = $\frac{\sum Infected\;plants}{\sum plants}\times 100$. The percent incidence was scored using the diseases rating scale in which fields with 0% incidence were recorded as healthy; >0–25% as low incidence; >25–50% as medium incidence; >50–75% as high incidence; and >75–100% as very high incidence. Whitefly abundance was calculated by counting whiteflies on the top five fully opened leaves. The mean of the whiteflies per plant was calculated as the total number of whiteflies recorded on 30 plants divided by 30.

### 4.4. Molecular Detection

Cassava leaf samples were utilized for the total DNA extraction using the CTAB protocol [47]. DNA concentration was carried out using a NanoDrop™ 2000 spectrophotometer (Thermo Fisher Scientific Inc., Waltham, MA, USA) by adjusting the equipment to 150 ng/μL. Previous studies noted that the most cumbersome CMBs in smallholder cassava cultivation systems in Sierra Leone were ACMV and East African cassava mosaic virus (EACMV) [29]. For detection of the ACMV-like and EACMV-like viruses, the DNA samples were subjected to PCR using specific primers (Table 3). The positive samples for the EACMV-like virus were subjected to another round of PCR using specific primers to detect EACMVCM. The PCR mix was prepared in a final volume of 25 μL using 20.9 μL of molecular biology grade water, 2.5 µL of 10× reaction buffer, 0.5 µL of 10 mM dNTPs, 0.5 µL of 10 µM of each primer, 0.1 µL of 5 U/µL of Maximo Taq DNA polymerase (GeneON), and 150 ng DNA template of each sample. DNA amplification was carried out in a SimpliAmp thermal cycler (Life Technologies Holdings Pte Ltd., Marsiling Industrial Estate, Marsiling, Singapore). The PCR temperature profile was set at 94 °C for 4 or 5 min for initial denaturation, followed by 35 cycles of amplification at 94 °C for 45 or 60 s, 55 °C for 45 or 60 s, and 72 °C for 55 or 60 s (depending on primers). The final elongation step was performed at 72 °C for 7 or 10 min. The PCR-amplified products were subjected to 1% agarose gel electrophoresis and then stained with ethidium bromide. The electrophoresis was performed at 100 V and the gel was visualized using a Compact Digimage System, UVDI series (MS major science, Saratoga, CA, USA).

### 4.5. Data Analysis

#### 4.5.1. Phenotypic Analysis

The phenotypic traits recorded were subjected to analysis in R software v. 3.6.1 (R Development Core Team, Copenhagen Business School Solbjerg Plads 3, 2000, Frederiksberg, Denmark). The normality of the phenotypic variables was estimated through the Shapiro–Wilk test. The generalized linear model was used for variables that were not distributed according to the normal distribution. The difference in the number of whiteflies per plant between regions and in the severity score of CMD between regions were assessed using the generalized linear model. The map of Sierra Leone showing the regions where surveys were conducted in 2024 was developed using QGIS software v. 2.18.26 (https://qgis.org/downloads/ (accessed on 21 October 2016)).

#### 4.5.2. Molecular Analysis

About 50 EACMV-like positive sample PCR products were sequenced in forward and reverse orientations at the Macrogen Meibergdreef 57 1105 BA, Amsterdam, the Netherlands, Europe, using the Sanger et al. [50] method. This was followed by assembling and editing contigs through the Geneious Prime^®^ 2022.2.1. (Biomatters Ltd., Auckland, New Zealand) software. Consensus sequence obtained from forward and reverse sequences for each sample was subjected to BLASTn in NCBI for preliminary species assignment and pairwise sequence comparison [51]. Sequence alignments with representative isolates of begomoviruses was carried out through the ClustalW alignment method in MEGA X software v. 10.2.5 [52].

## 5. Conclusions

This study identified and mapped the distribution of CMD-associated viruses (begomoviruses) in farmers’ fields in Sierra Leone that could be exploited for effective phytosanitation strategies to mitigate their effect on crop yield loss. The cassava farms in the east region, north region, northwest region, and western areas were more susceptible to disease attack due to the heavy utilization of infected cutting planting materials. The spread of the disease is linked to the use of infected planting materials, mainly through stem cuttings. This is the first report on the spread of EACMV-Ug infecting local cassava varieties in smallholder farms across south, north, and northwest regions of Sierra Leone. The identification of EACMV-Ug in four districts, three of which sharing border with Guinea, presents a serious threat to cassava production in the country. Findings suggest timely implementation of rapid mitigating strategies by national and regional cassava stakeholders in Sierra Leone and the subregion. Routine cassava farm disease status assessment should be exploited to ascertain the spread of EACMV-Ug. Awareness campaigns should be carried out to various stakeholders, including farmers, extension officers, and policymakers, on the importance of CMD, identification of the disease symptoms, and best practices to mitigate its spread. Efficient disease response and phytosanitation (i.e., seed certification and deployment of resistant genotypes) strategies should be implemented to limit the spread of EACMV-Ug in Sierra Leone and the ECOWAS region.

## Figures and Tables

**Table 1:** Viral strains associated with CMD in Sierra Leone.

Number of Samples	Status of Samples	Positive Samples				Negative Samples
		Detection of ACMV	Detection of EACMV	Detection of ACMV/EACMV	Detection of EACMVCM Virus	
	320 CMD	230	4	58	43	28
426	106 Symptomless	13	0	6	5	87
	Total	243	4	64	48	115

Values in brackets are positive single infection of ACMV after delineating for EACMV, EACMVCM, and mixed infections. Total positive samples = 311 and total negative samples = 115.

**Table 2:** Distribution of begomoviruses across regions of Sierra Leone.

Region	Tested Samples	Positive Samples	ACMV-Single Infection		EACMV-Single Infection		Mixed Infection (ACMV/EACMV)		EACMVCM Virus	
			n	%	n	%	n	%	n	%
South	112	73	47	64.4	0	0.0	26	35.6	26	100.0
East	81	68	53	77.9	1	1.5	14	20.6	8	53.3
North	110	81	71	87.7	0	0.0	10	12.3	6	60.0
Northwest	79	60	45	75.0	3	5.0	12	20.0	8	53.3
West	44	29	27	93.1	0	0.0	2	6.9	0	0.0
Total	426	311	243	78.1	4	1.3	64	20.6	48	70.6

n = sample size; % = percent.

**Table 3:** Details of primer pairs utilized for detection of virus species.

Primer	Sequence (5′-3′)	Target Region	Expected Size (bp)	Virus Species	Reference
JSP001	ATGTCGAAGCGACCAGGAGAT	DNA-A (CP)	783	ACMV	Pita et al. [37]
JSP002	TGTTTATTAATTGCCAATACT				
ACMBVF	TCGGGAGTGATACATGCGAAGGC	DNA-B (BV1/BC1)	628	ACMV	Matic et al. [48]
ACMBVR	GGCTACACCAGCTACCTGAAGCT				
WAVE-508F	AAGGCCCATGTAAGGTCCAG	AV1/AC3	800	ACMV	WAVE
WAVE-1307R	GAAGGAGCTGGGGATTCACA				
WAVE-177F	GATCTGCGGGCCTATCGAAT	BV1	800	ACMV	WAVE
WAVE-197R	TTCACGCTGTGCAATACCCT				
WAVE-370F	ACAGCCCATACAGGAACCGT	AV1/AC3	1000	ACMV	WAVE
WAVE-1369R	CGACCATTCCTGCTGAACCA				
WAVE-982F	TTCGTGTCATCTGCAGGAGA	BV1/BC1	800	ACMV	WAVE
WAVE-1781R	GTACCATGGCAGCTGCTGTA				
JSP001	ATGTCGAAGCGACCAGGAGAT	DNA-A (CP)	780	EACMV	Pita et al. [37]
JSP003	CCTTTATTAATTTGTCACTGC				
CMBRepF	CRTCAATGACGTTGTACCA	DNA-A (AC1)	650	EACMV	Alabi et al. [49]
EACMVRepR	GGTTTGCAGAGAACTACATC				
WAVE-EA1875F	TGTACCAGGCGTCGTTTGAA	AC1	800	EACMVCM	WAVE
WAVE-E2674R	TGTCCCCCGATCCAAAACG				
WAVE-EB1869F	TTCCAAGGGGAGGGTTCTGA	BC1	800	EACMV	WAVE

## Data Availability

For ethical reasons, the corresponding author can make data from this research available upon request.

## Abbreviations

This study utilized the following abbreviations:

ACMV	African cassava mosaic virus
CMD	Cassava mosaic disease
CTAB	Cetyltrimethylammonium bromide
DNA	Deoxyribonucleic acid
EACMV	East African cassava mosaic virus
MEGA	Molecular Evolutionary Genetics Analysis
PCR	Polymerase chain reaction

## References

[1] The WIRE (2020). Why Cassava Has So Much Potential in Sub-Saharan Africa. https://thewire.in/agriculture/food-security-climate-change-cassava.

[2] The Food and Agriculture Organization Statistics FAOSTAT (2020). https://www.fao.org/faostat/en/#data/QCL.

[3] Tarawali G., Ilona P., Ojiako I.A., Iyangbe C., Ogundijo D.S., Asumugha G., Udensi U.E. (2013). A Comprehensive Training Module on Competitive Cassava Production.

[4] Storey H.H. (1936). Virus diseases of East African plants: VI-A progress report on studies of the diseases of cassava. East Afr. Agric. J..

[5] Winter S., Koerbler M., Stein B., Pietruszka A., Paape M., Butgereitt A. (2010). Analysis of cassava brown streak viruses reveals the presence of distinct virus species causing cassava brown streak disease in East Africa. J. Gen. Virol..

[6] Owor B., Legg J.P., Okao-Okuja G., Obonyo R., Ogenga-Latigo M.W. (2004). The effect of cassava mosaic geminiviruses on symptom severity, growth and root yield of a cassava mosaic virus disease-susceptible cultivar in Uganda. Ann. Appl. Biol..

[7] Legg J.P., Owor B., Sseruwagi P., Ndunguru J. (2006). Cassava mosaic virus disease in east and central Africa: Epidemiology and management of a regional pandemic. Adv. Virus Res..

[8] Fauquet C.M., Briddon R.W., Brown J.K., Moriones E., Stanley J., Zerbini M., Zhou X. (2008). Geminivirus strain demarcation and nomenclature. Arch. Virol..

[9] Samura A.E., Massaquoi F.B., Mansaray A., Kumar P.L., Koroma J.P.C., Fomba S.N., Dixon A. (2014). Status and diversity of the cassava mosaic disease causal agents in Sierra Leone. Int. J. Agric. For..

[10] Chi Y., Pan L.L., Bouvaine S., Fan Y.Y., Liu Y.Q., Liu S.S., Seal S., Wang X.W. (2020). Differential transmission of Sri Lankan cassava mosaic virus by three cryptic species of the whitefly *Bemisia tabaci* complex. Virology.

[11] Njoroge M.K., Mutisya D.L., Miano D.W., Kilalo D.C. (2017). Whitefly species efficiency in transmitting cassava mosaic and brown streak virus diseases. Cogent Biol..

[12] Maruthi M.N., Hillocks R.J., Mtunda K., Raya M.D., Muhanna M., Kiozia H., Rekha A.R., Colvin J., Thresh J.M. (2005). Transmission of cassava brown streak virus by *Bemisia tabaci* (Gennadius). J. Phytopathol..

[13] Chikoti P.C., Tembo M., Chisola M., Ntawuruhungu P., Ndunguru J. (2015). Status of cassava mosaic disease and whitefly population in Zambia. Afr. J. Biotechnol..

[14] Thresh J.M., Cooter R.J. (2005). Strategies for controlling cassava mosaic virus disease in Africa. Plant Pathol..

[15] Sseruwagi P., Sserubombwe W.S., Legg J.P., Ndunguru J., Thresh J.M. (2004). Methods of surveying the incidence and severity of cassava mosaic disease and whitefly vector populations on cassava in Africa: A review. Virus Res..

[16] Crespo-Bellido A., Hoyer J.S., Dubey D., Jeannot R.B., Duffy S. (2021). Interspecies recombination has driven the macroevolution of cassava mosaic begomoviruses. J. Virol..

[17] Maruthi M.N., Seal S., Colvin J., Briddon R.W., Bull S.E. (2004). East African cassava mosaic Zanzibar virus—A recombinant begomovirus species with a mild phenotype. Arch. Virol..

[18] Monde G., Walangululu J., Winter S., Bragard C. (2010). Dual infection by cassava begomoviruses in two leguminous species (Fabaceae) in Yangambi, northeastern Democratic Republic of Congo. Arch. Virol..

[19] Patil B.L., Fauquet C.M. (2009). Cassava mosaic geminiviruses: Actual knowledge and perspectives. Mol. Plant Pathol..

[20] Ndunguru J., Legg J.P., Aveling T.A.S., Thompson G., Fauquet C.M. (2005). Molecular biodiversity of cassava begomoviruses in Tanzania: Evolution of cassava geminiviruses in Africa and evidence for East Africa being a center of diversity of cassava geminiviruses. Virol. J..

[21] Ariyo O.A., Koerbler M., Dixon A.G.O., Atiri G.I., Winter S. (2005). Molecular variability and distribution of cassava mosaic begomoviruses in Nigeria. J. Phytopathol..

[22] Were H.K., Winter S., Maiss E. (2003). Distribution of begomoviruses infecting cassava in Africa. J. Plant Pathol..

[23] Ogero K.O., Mburugu G.N., Mwangi M., Ombori O., Ngugi M. (2012). In vitro micropropagation of cassava through low-cost tissue culture. Asian J. Agric. Sci..

[24] Nassar N.M.A., Ortiz R. (2007). A review on cassava improvement: Challenges and impacts. J. Agric. Sci..

[25] Bakelana Z., Boykin L.M., Mahungu N., Mavila N., Magole M., Nduandele N., Makuati L., Ndomateso T., Monde G., Pita J. (2019). First report and preliminary evaluation of cassava root necrosis in Angola. Int. J. Agric. Environ. Biores..

[26] Legg J.P., Jeremiah S.C., Obiero H.M., Maruthi M.N., Ndyetabula I., OkaoOkuja G., Bouwmeester H., Bigirimana S., Tata-Hangy W., Gashaka G. (2011). Comparing the regional epidemiology of the cassava mosaic and cassava brown streak virus pandemics in Africa. Virus Res..

[27] Samura A.E., Kanteh S.M., Norman J.E., Fomba S.N. (2016). Integrated pest management options for the cassava mosaic disease in Sierra Leone. Int. J. Agric. Innov. Res..

[28] Sesay J.V., Ayeh K.O., Norman P.E., Acheampong E. (2016). Shoot nodal culture and virus indexing of selected local and improved genotypes of cassava (*Manihot esculenta*) from Sierra Leone. Int. J. Biotechnol. Mol. Biol. Res..

[29] Sesay J.V., Lebbie A., Wadsworth R., Nuwamanya E., Bado S., Norman P.E. (2023). Genetic structure and diversity study of cassava (*Mannihot esculenta*) germplasm for African cassava mosaic disease and fresh storage root yield. Open J. Genet..

[30] Mivedor A.S., Dansou-Kodjo K.A., Adjata D.K., Pita J.S. (2020). Identification and incidence of cassava mosaic begomoviruses in Togo. Asian J. Plant Pathol..

[31] Chikoti P.C., Peter S., Mulenga R.M., Tembo M. (2019). Cassava mosaic disease: A review of a threat to cassava production in Zambia. J. Plant Pathol..

[32] Fondong V.N., Pita J.S., Rey M.E.C., de Kochko A., Beachy R.N., Fauquet C.M. (2000). Evidence of synergism between African cassava mosaic virus and a new double-recombinant geminivirus infecting cassava in Cameroon. J. Gen. Virol..

[33] Ogbe F.O., Thottappilly G., Dixon A.G., Atiri G.I., Mignouna H.D. (2003). Variants of East African cassava mosaic virus and its distribution in double infections with African cassava mosaic virus in Nigeria. Plant Dis..

[34] Alicai T., Omongo C.A., Maruthi M.N., Hillocks R.J., Baguma Y., Kawuki R., Bua A., Otim-Nape G.W., Colvin J. (2007). Re-emergence of cassava brown streak disease in Uganda. Plant Dis..

[35] Njoroge M.K., Kilalo D.C., Miano D.W., Mutisya D.L. (2016). Whiteflies species distribution and abundance on cassava crop in different agro-ecological zones of Kenya. J. Entomol. Zool. Stud..

[36] Combala M., Pita J.S., Gbonamou M., Samura A.E., Amoakon W.J.-L., Kouakou B.S.M., Onile-ere O., Sawadogo S., Eboulem G.R., Otron D.H. (2024). An alarming eastward front of cassava mosaic disease in development in West Africa. Viruses.

[37] Pita J.S., Fondong V.N., Sangare A., Otim-Nape G.W., Ogwal S., Fauquet C.M. (2001). Recombination, pseudorecombination and synergism of geminiviruses are determinant keys to the epidemic of severe cassava mosaic disease in Uganda. J. Gen. Virol..

[38] Valam-zango A., Zinga I., Hoareau M., Mvila A.C., Semballa S., Lett J.M. (2015). First report of cassava mosaic geminiviruses and the Uganda strain of East African cassava mosaic virus (EACMV-UG) associated with cassava mosaic disease in Equatorial Guinea. New Dis. Rep..

[39] Doyle J.J., Doyle J.L. (1987). A rapid DNA isolation procedure for small quantities of fresh leaf tissue. Phytochem. Bull..

[40] Kumar P.L., Cuervo M., Kreuze J.F., Muller G., Kulkarni G., Kumari S.G., Massart S., Mezzalama M., Alakonya A., Muchugi A. (2021). Phytosanitary interventions for safe global germplasm exchange and the prevention of transboundary pest spread: The role of CGIAR germplasm health units. Plants.

[41] Harimalala M., Chiroleu F., Giraud-Carrier C., Hoareau M., Zinga I., Randriamampianina J.A., Velombola S., Ranomenja-nahary S., Andrianjaka A., Reynaud B. (2014). Molecular epidemiology of cassava mosaic disease in Madagascar. Plant Pathol..

[42] Eni A.O., Efekemo O.P., Onile-ere O.A., Pita J.S. (2021). South west and north central Nigeria: Assessment of cassava mosaic disease and field status of African cassava mosaic virus and East African cassava mosaic virus. Ann. Appl. Biol..

[43] Busogoro J.P., Masquellier L., Kummert J., Dutrecq O., Lepoivre P., Jijakli M.H. (2008). Application of a simplified Molecular protocol to reveal mixed infections with begomoviruses in cassava. J. Phytopathol..

[44] Zhou X., Robinson D.J., Harrison B.D. (1998). Types of variation in DNA—A among isolates of East African cassava mosaic virus from Kenya, Malawi and Tanzania. J. Gen. Virol..

[45] Fargene D., Colon L.T., Bouveau R., Fauquet C. (1996). Components of resistance of cassava to African cassava mosaic virus. Eur. J. Plant Pathol..

[46] IITA (International Institute of Tropical Agriculture) (1990). Cassava in Tropical Africa, A Reference Manual.

[47] Permingeat H.R., Romagnoli M.V., Sesma J.I., Vallejos R.H. (1998). A simple method for isolating DNA of high yield and quality from cotton (shape *Gossypium hirsutum* L.) leaves. Plant Mol. Biol. Rep..

[48] Matic S., da Cunha A.T.P., Thompson J.R., Tepfer M. (2012). An analysis of viruses associated with cassava mosaic disease in three Angolan provinces. J. Plant Pathol..

[49] Alabi O.J., Kumar P.L., Naidu R.A. (2008). Multiplex PCR method for the detection of African cassava mosaic virus and East African cassava mosaic Cameroon virus in cassava. J. Virol. Methods.

[50] Sanger F., Nicklen S., Coulson A.R. (1977). DNA sequencing with chain-terminating inhibitors. Proc. Nat. Acad. Sci. USA.

[51] Bao Y., Chetvernin V., Tatusova T. (2014). Improvements to pairwise sequence comparison (PASC): A genome-based web tool for virus classification. Arch. Virol..

[52] Kumar S., Stecher G., Li M., Knyaz C., Tamura K. (2018). MEGA X: Molecular evolutionary genetics analysis across computing platforms. Mol. Biol. Evol..

